# Gas Chromatography Combustion Isotope Ratio Mass Spectrometry for Improving the Detection of Authenticity of Grape Must

**DOI:** 10.1021/acs.jafc.9b05952

**Published:** 2020-02-03

**Authors:** M. Perini, L. Strojnik, M. Paolini, F. Camin

**Affiliations:** † Fondazione Edmund Mach, Via Edmund Mach 1, 38010 San Michele all’Adige, Italy; ‡ Department of Environmental Sciences, Jožef Stefan Institute, 1000 Ljubljana, Slovenia; § Jožef Stefan International Postgraduate School, 1000 Ljubljana, Slovenia; ⊥ Center Agriculture Food Environment (C3A), University of Trento, 38010 San Michele all’Adige, Italy

**Keywords:** GC−C−IRMS, stable isotope analysis, proline, *myo*- and *scyllo*-inositols, chaptalization, grape must

## Abstract

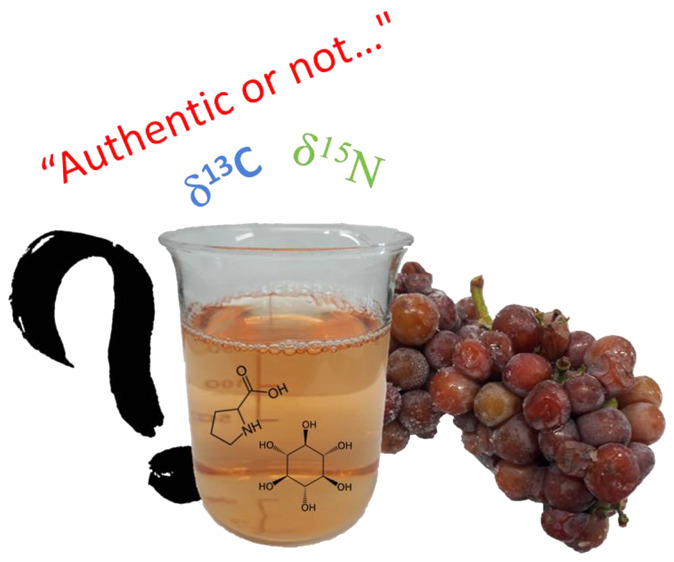

Since ancient times, grape must and wine have been considered one of the most sophisticated matrices and, in the last few years, the continuous rise in volumes and prices of grapes and wine has encouraged fraud and adulteration in the oenological field. One of the most common adulterations is sugar addition to grape must in the form of cane or beet sugar or syrup coming from vegetable sources, such as cereals or fruits. Since 1990, the International Organisation of Vine and Wine (OIV) has issued specific official isotopic methods to fight against this practice, but they are not always effective. With the aim to develop a new method able to identify sugar addition, we compared the δ^13^C value of sugar extracted from grape must analyzed by elemental analyzer/isotope ratio mass spectrometry (EA-IRMS) to the δ^13^C value of proline analyzed by gas chromatography-combustion isotope ratio mass spectrometry (GC–C–IRMS), after extraction and derivatization. δ^13^C and δ^15^N of proline have also been tested as potential geographical markers. In addition, the carbon isotopic composition of two characteristic grape must sugars (*myo*- and *scyllo*-inositols) was measured by GC–C–IRMS, after derivatization, to identify the illegal correction of their concentration. On the basis of the obtained results we can conclude that the compound-specific isotope analysis represents a novel analytical tool to support and improve certification and control procedures.

## 1 Introduction

According to the International Organisation of Vine and Wine (OIV) definition wine is the beverage resulting exclusively from the partial or complete alcoholic fermentation of fresh grapes, whether crushed or not, or grape must that is the liquid product obtained naturally or by physical processes from fresh grapes.^1^ OIV has established for wine a unique minimum alcoholic strength of 8.5 vol %, with the flexibility to be reduced to 7 vol %, to guarantee its stability.^2^ To reach this limit the addition of sources of sugar to grape must before fermentation is allowed. While the addition of rectified must to grape must or wine before or during fermentation to increase the alcoholic strength is always permitted in all of the countries, the use of beet sugar and cane sugar is legal only for specific winegrowing regions and vintages, e.g., in Brazil, Canada, Chile, China, France, Germany, Japan, New Zealand, Switzerland, United Kingdom, and the United States.^3^ In Italy, the addition of exogenous sugar (beet or cane) is forbidden and constitutes a fraud (chaptalization) by unscrupulous wine producers to increase profit.^4^

In 1990, the European Commission (EC) adopted isotopic methods as the first official analytical methods (now OIV MA-AS-311-05, MA-AS-312-06, and MA-AS2-12) to detect and combat these types of grape must and wine frauds.^5^ They are based on the analysis of the isotopic ratios of hydrogen (D/H) and carbon (^13^C/^12^C, expressed as δ^13^C) in ethanol distilled from wine and after must fermentation. Unfortunately, during the last few decades, adulterations became increasingly sophisticated;^6^ thus, the development of even more powerful analytical methods for must and wine authentication is a great challenge.

The direct stable isotope ratio analysis of single chemical compounds, normally measured by a gas chromatography combustion isotope ratio mass spectrometry (GC–C–IRMS) technique, provides a means of obtaining a more in-depth understanding with respect to the traditional analysis of bulk products.^7^ Examples of application are reported for single amino acids in wheat and durum wheat samples, to discriminate between organic and conventional agricultural practices,^8^ vanillin, to discriminate between natural and synthetic samples,^9^ fatty acid, to identify the adulteration of high-value oils with cheaper oils,^6^ wine volatile compounds, to reassess the water status in vineyards.^10^ In relation to wine, Spitzke at al. developed a GC–C–IRMS method to analyze ^13^C/^12^C of higher alcohols, for example, 2-methylpropan-1-ol, 2- and 3-methylbutan-1-ol, butan-2,3-diol, 2-phenyl-1-ethanol, and glycerol.^11^ From the correlation of δ^13^C of higher alcohol compounds (such as 2-/3-methylbutan-1-ol) with that of wine ethanol (*R*^2^ = 0.829) they were able to improve the detection of chaptalization. Other groups,^12,13^ have also investigated the isotopic composition of ethanol and glycerol by GC–C–IRMS as alternative techniques to determine adulteration of wines.

This paper illustrates two studies that propose new specific compound methods to improve the identification of grape must authenticity.

In the first study we compared, for the first time, δ^13^C of the sugar fraction extracted from grape must using the official method UNI ENV 12140:1997 with δ^13^C of proline after extraction and derivatization.^14^ Among the amino acids the most abundant in wine and grapes is proline with a content ranging between 30 and 85%,^15^ and this makes it possible to obtain a sufficient quantity of it for derivatization and analysis. Moreover, yeast does not require proline as a nitrogen source and it is therefore maintained in wine. The isotopic ratio of nitrogen (^15^N/^14^N, expressed as δ^15^N) was also analyzed using proline as an additional marker to trace the geographical origin of grapes. As reported by Paolini et al.,^8^ the isotopic ratio of nitrogen could be a useful additional marker because, different from H, O, and C, nitrogen in grape compounds derives directly from the soil and therefore the factors affecting its isotopic variability are different from those affecting the other three isotopic ratios.^16^

In the second study we tested two characteristic grape must sugars (*myo*- and *scyllo*-inositols) after derivatization to see if the analysis of their δ^13^C values could be useful in identifying the illegal correction of their concentration in concentrated and rectificated grape must (CRM). These polyalcohols originate in the grape berry and their quantification has already been proposed by Monetti et al. to control the authenticity of the CRM because they are not retained by the resins used for the concentration process and are not present in other purified commercial sugars.^17^ A minimum content of *myo*-inositol (750 mg/kg of sugar) and a *myo*- and *scyllo*-inositol ratio of 20 or less have been suggested as authenticity indexes.^17^ In Regulation 479/2008 this proposal was only partially adopted and today official CRM controls focus on the presence of *myo*-inositol alone, without considering its commercial availability, its levels in musts, and the relationship between the two isomers.^18^ There is the suspect that fraudsters correct the concentration of these two polyalcohols, in particular *myo*-inositol, by adding commercially pure *myo*- and *scyllo*-inositols to fake grape must concentrate originated from other fruits (e.g., date or tapioca) or from a mix of sugars. The δ^13^C variability of authentic and fake polyalcohols has been explored in this study and tested on samples coming from the market, to verify their validity as fraud detectors.

## 2 Materials and Methods

### 2.1 Reagents and Solutions

Proline (≥98%), *myo*-inositol (≥99%), *scyllo*-inositol (≥98%), and cation-exchange resin (Amberlite IR120) were purchased from Sigma-Aldrich. All other solvents (acetone, dichloromethane, ethanol, ethyl acetate, and isopropanol) and reagents [acetic anhydride, silylating agent hexamethyldisilazane (HMDS) + trimethylchlorosilane (TMCS) + pyridine 3:1:9, and triethylamine] used were of analytical grade and purchased from Sigma-Aldrich and VWR.

### 2.2 Sampling

#### 2.2.1 Italian Grape Must, First Study

A total of 59 authentic Italian grape musts were sampled during 2016 (*N* = 36) and 2017 (*N* = 23) harvests (Table 1). From one to five samples were collected from 15 different Italian regions (Abruzzo, Campania, Emilia Romagna, Friuli Venezia Giulia, Lazio, Liguria, Lombardy, Marche, Piedmont, Puglia, Sardinia, Sicily, Tuscany, Trentino Alto Adige, Umbria, and Veneto), at the usual technological harvest time (early, medium, and late harvesting) (Figure S1 of the Supporting Information). A total of 25 different grape varieties were considered to describe natural variability (Table 1). The sampling was supervised by the technicians of the Edmund Mach Foundation (San Michele all’Adige, Italy), who personally followed all of the harvesting and grape-crushing stages for grape must production (as part of the Italian Project “Climaitalia2020”). Proline was extracted from all of the samples, derivatized, and subjected to the analysis of δ^13^C and δ^15^N.

**Table 1:** Experimental Values of the Isotopic Composition of δ^13^C and δ^15^N of Proline, δ^13^C of the Sugar Fraction, and Variation between the Two δ^13^C

number	year of harvest	Italian region	variety of grape	δ^15^N proline (‰, vs AIR)	δ^13^C proline (‰, vs V-PDB)	δ^13^C sugar (‰, vs V-PDB)	δ^13^C sugar – δ^13^C proline
1	2016	Veneto	Oseleta	5.6	–24.2	–24.7	–0.5
2	2016	Veneto	Corvinone	4.2	–25.2	–24.9	0.3
3	2016	Veneto	Manzoni	4.7	–27.0	–28.1	–1.1
4	2016	Veneto	Chardonnay	4.8	–27.0	–26.5	0.5
5	2016	Friuli VG	Sauvignon	0.2	–27.6	–27.7	–0.1
6	2016	Friuli VG	Malvasia	0.0	–29.0	–28.2	0.8
7	2016	Piedmont	Nebbiolo	0.6	–28.8	–27.1	1.7
8	2016	Piedmont	Nebbiolo	–0.5	–28.8	–27.4	1.4
9	2016	Piedmont	Nebbiolo	–0.1	–28.8	–27.6	1.2
10	2016	Piedmont	Nebbiolo	–0.3	–26.2	–24.7	1.5
11	2016	Piedmont	Riesling	1.6	–27.8	–26.2	1.6
12	2016	Lombardy	Pinot	4.9	–27.4	–28.2	–0.8
13	2016	Lombardy	Chardonnay	0.5	–28.6	–27.6	1.0
14	2016	Emilia Romagna	Barbera	–1.5	–25.8	–26.1	–0.3
15	2016	Emilia Romagna	Cabernet	–2.5	–26.6	–26.6	0.0
16	2016	Toscany	Sangiovese	4.7	–25.8	–25.7	0.1
17	2016	Toscany	Sangiovese	4.0	–26.2	–25.6	0.6
18	2016	Lazio	Syrah	2.6	–26.2	–25.7	0.5
19	2016	Lazio	Vermentino	0.6	–27.0	–26.0	1.0
20	2016	Lazio	Malvasia	2.4	–25.0	–26.1	–1.1
21	2016	Lazio	Montepulciano	0.8	–28.8	–28.1	0.7
22	2016	Marche	Verdicchio	4.4	–28.8	–27.2	1.6
23	2016	Marche	Verdicchio	6.5	–30.0	–28.4	1.6
24	2016	Marche	Montepulciano	4.5	–29.4	–28.7	0.7
25	2016	Marche	Sangiovese	3.7	–29.8	–28.8	1.0
26	2016	Marche	Verdicchio	2.8	–28.4	–27.7	0.7
27	2016	Abruzzo	Trebbiano	3.2	–28.6	–27.3	1.3
28	2016	Umbria	Grechetto	3.7	–27.6	–27.6	0.0
29	2016	Umbria	Sagrantino	2.4	–28.8	–28.9	–0.1
30	2016	Campania	Aglianico	4.5	–25.2	–25.9	–0.7
31	2016	Puglia	Primitivo	6.5	–26.0	–25.4	0.6
32	2016	Puglia	Primitivo	5.0	–26.0	–25.6	0.4
33	2016	Sicily	Insolia	3.7	–24.2	–25.7	–1.5
34	2016	Sicily	Insolia	2.0	–26.4	–25.4	1.0
35	2016	Sardinia	Vermentino	6.4	–26.6	–25.6	1.0
36	2016	Sardinia	Monica	9.8	–24.2	–23.3	0.9
			mean	3.0	-27.2	-26.7	0.5
			SD	2.6	1.6	1.4	0.8
			minimum	–2.5	–30.0	–28.9	–1.5
			maximum	9.8	–24.2	–23.3	1.7
1	2017	Trentino AA	Muller Thurgau	8.0	–28.4	–28.7	–0.3
2	2017	Trentino AA	Pinot Grigio	6.5	–26.4	–27.5	–1.1
3	2017	Trentino AA	Kerner	9.2	–27.2	–28.4	–1.2
4	2017	Veneto	Chardonnay	2.8	–27.0	–26.6	0.4
5	2017	Veneto	Chardonnay	1.2	–24.6	–25.8	–1.2
6	2017	Friuli VG	Chardonnay	–1.0	–28.4	–27.9	0.5
7	2017	Piedmont	Riesling	2.4	–26.2	–27.2	–1.0
8	2017	Piedmont	Nebbiolo	0.4	–25.4	–26.7	–1.3
9	2017	Piedmont	Nebbiolo	2.9	–27.8	–27.4	0.4
10	2017	Piedmont	Nebbiolo	4.5	–27.2	–26.8	0.4
11	2017	Piedmont	Nebbiolo	5.4	–25.8	–24.9	0.9
12	2017	Lombardy	Pinot	5.8	–27.0	–26.8	0.2
13	2017	Toscany	Sangiovese	2.6	–23.6	–23.4	0.2
14	2017	Lazio	Malvasia	5.2	–27.0	–26.8	0.2
15	2017	Lazio	Montepulciano	2.6	–28.0	–27.7	0.3
16	2017	Marche	Verdicchio	6.0	–27.0	–25.6	1.4
17	2017	Marche	Verdicchio	8.0	–27.2	–25.5	1.7
18	2017	Marche	Verdicchio	3.7	–24.6	–26.2	–1.6
19	2017	Abruzzo	Montepulciano	4.1	–24.6	–24.6	0.0
20	2017	Abruzzo	Montepulciano	6.2	–23.4	–23.0	0.4
21	2017	Abruzzo	Montepulciano	6.2	–25.2	–23.8	1.4
22	2017	Umbria	Grechetto	5.4	–24.4	–25.4	–1.0
23	2017	Campania	Aglianico	2.1	–24.4	–24.2	0.2
			mean	4.4	-26.1	-26.1	0.0
			SD	2.6	1.5	1.6	0.9
			minimum	–1.0	–28.4	–28.7	–1.6
			maximum	9.2	–23.4	–23.0	1.7

#### 2.2.2 Rectified Concentrated Grape Must, Second Study

A total of 9 authentic CRM of the 2018 harvest were collected (Table 2). In addition, 7 commercial CRM samples from Italy and Spain were also considered.

**Table 2:** Experimental Values of the Isotopic Composition of δ^13^C of *myo*- and *scyllo*-Inositol and Variation between the Two δ^13^C in Authentic Samples of CRM and in Samples from the Market

	sample	geographical origin	δ^13^C *scyllo*-inositol (‰, vs V-PDB)	δ^13^C *myo*-inositol (‰, vs V-PDB)	δ^13^C *scyllo* – δ^13^C *myo*	*myo*-inositol (mg/kg)	*scyllo*-inositol (mg/kg)	ratio (*myo*-inositol/*scyllo*-inositol)
authentic CRM samples	A	Puglia	–28.6	–28.2	–0.4	1650	260	6
	B	Italy	–28.6	–26.2	–2.4	1420	230	6
	C	Italy	–28.6	–29.4	0.8	1300	150	9
	D	Italy	–28.2	–29.4	1.2	1350	210	6
	E	Italy	–27.0	–27.4	0.4	1470	250	6
	F	Italy	–27.0	–28.2	1.2	1760	270	7
	G	Italy	–26.6	–29.0	2.4	1310	200	7
	H	Italy	–25.0	–25.8	0.8	1510	180	8
	I	Sicily	–25.0	–26.2	1.2	1790	210	9
		mean	–27.2	–27.8	0.6			
		SD	1.5	1.4	1.3			
		minimum	–28.6	–29.4	–2.4			
		maximum	–25.0	–25.8	2.4			
market’s CRM	L	Italy	–23.4	–25.8	2.4	1010	160	6
	M	Italy	–22.2	–27.8	5.6	1550	220	7
	N	Italy	–21.8	–27.8	6.0	1340	190	7
	O	Spain	–21.8	–27.4	5.6	2020	270	7
	P	Spain	–19.0	–23.4	4.4	820	110	7
	Q	Spain	–25.0	–21.0	–4.0	800	110	7
	R	Spain	–25.8	–25.8	0.0	930	130	7

### 2.3 Elemental Analysis Isotope Ratio Mass Spectrometry (EA–IRMS) Analysis

#### 2.3.1 Extraction of the Sugar Fraction

The sugar fraction was extracted using the official method UNI ENV 12140. In brief, the solid fraction of the must (50 mL) was removed by centrifugation at 1400*g* for 10 min. A total of 2 g of powdered calcium hydroxide was added to the supernatant liquid, and the solution was heated in a bath at 90 °C for 3 min. The precipitate was separated by centrifugation of the hot solution (3 min at 1400 g), and the supernatant liquid was acidified with 0.1 M sulfuric acid to obtain a pH value of approximately 5. After a night in the refrigerator (4 °C), the supernatant liquid was freeze-dried to obtain the sugar fraction.

#### 2.3.2 EA–IRMS Analysis of δ^13^C

The δ^13^C value of sugar fraction samples was measured using an elemental analyzer (Flash EA 1112, Thermo Scientific, Bremen, Germany), coupled with IRMS (DELTA V, Thermo Scientific) through a ConFlo IV dilutor device (Thermo Finningan, Bremen, Germany).

### 2.4 GC–C–IRMS Analysis

#### 2.4.1 Isolation and Derivatization of Proline

The grape must sample was adjusted to pH 2.3 with 0.01 M HCl, and 100 μL of a norleucine solution (8 mg/mL in 0.1 M HCl) was added as an internal standard. Amino acids were isolated using an Amberlite IR120 cation-exchange resin, previously saturated with H^+^ at all exchange sites as reported by Takano et al.^19^ A total of 5 mL of grape must sample was passed through the resin column and washed with water. Finally, amino acids were eluted with NH_4_OH (10%, w/w) and then dried under N_2_.^20^

Amino acids were analyzed after *N*-acetylisopropyl derivatization, following the method reported by Corr at al.^14^ Briefly, amino acids were esterified with 1 mL of acetyl chloride–isopropanol mixture (1:4, v/v) at 100 °C for 1 h and then acylated with 1 mL of acetic anhydride–triethylamine–acetone mixture (1:2:5, v/v/v) at 60 °C for 10 min. The reagents were evaporated under a gentle stream of nitrogen at room temperature, and 1 mL of saturated sodium chloride–water solution and 1 mL of ethyl acetate were added and then mixed vigorously. The organic layer was dried under nitrogen; residual water was removed with dichloromethane; and finally the derivatized amino acids were dissolved in ethyl acetate (200 mL).

#### 2.4.2 Derivatization and Quantification of *myo*- and *scyllo*-Inositols

Derivatization and quantification of *myo*- and *scyllo*-inositols in CRM were performed following the official method RESOLUTION OIV-OENO 419C-2015. Briefly, 5 g of CRM was weighted in a 50 mL volumetric flask, adding 1 mL of xylitol standard solution (10 000 mg/L in water) and then brought to volume with water. A total of 100 μL of the final solution was dried under a gentle stream of nitrogen, and 400 μL of the derivatization mixture (HMDS + TMCS + pyridine, 3:1:9) was added. The vial was closed and placed in the oven at 80 °C for 60 min.

*Myo*- and *scyllo*-inositols were quantified using an Agilent Intuvo 9000 GC–FID system, injecting 3 μL in split mode (1:10) into a 30 m HP-5MS Ultra Inert column (0.32 mm I.D. × 0.25 μm film thickness, Agilent) with H_2_ as the carrier gas (2 mL/min). The oven temperature was programmed starting at 100 °C, raised to 240 °C by 10 °C/min, then raised to 260 °C by 2 °C/min, finally raised to 310 °C by 50 °C/min, and held at this temperature for 5 min. The injector temperature was set at 270 °C.

#### 2.4.3 GC–C–IRMS Analysis of Proline

The δ^13^C and δ^15^N values of proline were determined using a Trace GC Ultra (GC IsoLink + ConFlo IV, Thermo Scientific) interfaced with IRMS (DELTA V, Thermo Scientific) and a single-quadrupole MS detector (ISQ Thermo Scientific). A total of 0.5 μL of each sample was injected in splitless mode, and a 60 m HP-INNOWAX capillary column (0.32 mm I.D. × 0.25 μm film thickness, Agilent) was used with He as the carrier gas (1.4 mL/min). The injector was at 250 °C, and the oven temperature program was set as follows: initially at 40 °C held for 2 min, ramped to 140 °C at 40 °C/min, followed by ramped to 180 °C at 2.5 °C/min, then ramped to 220 °C at 6 °C/min, and finally ramped to 250 °C at 40 °C/min for 15 min.

The eluted proline was combusted into N_2_ and CO_2_ in a combustion furnace reactor operated at 1030 °C. During δ^15^N analysis, a liquid nitrogen trap was added after the reactor to block CO_2_.

To monitor derivatization step and instrumental performance, a standard proline was derivatized and the δ^13^C and δ^15^N values were measured using GC–C–IRMS before and after each analytical run and compared to the isotopic composition measured directly by EA–IRMS (δ^13^C = −24.5‰, and δ^15^N = +1.1‰) without any derivatization step. Moreover, the isotopic value of the internal standard norleucine added to each sample was checked. Norleucine was chosen as internal standard because it is not naturally present in wine. The δ^15^N and δ^13^C values of pure norleucine (+14.0 and −27.6‰, respectively) were determined by using EA-IRMS, and the analytical run was accepted when the differences between GC–C–IRMS and EA–-IRMS values were, at most, ±1.0 and ±1.5‰, respectively for δ^15^N and δ^13^C.

#### 2.4.4 GC–C–IRMS Analysis of *myo*- and *scyllo*-Inositols

The δ^13^C values of *myo*- and *scyllo*-inositols were determined injecting 2 μL in splitless mode in a 30 m HP5-MS capillary column (0.32 mm I.D. × 1.00 μm film thickness, Agilent) with He as the carrier gas at 1.5 mL/min. The injector was at 250 °C, and the oven temperature program used is as follows: held for 20 min at 150 °C, increased to 220 °C at 10 °C/min, finally ramped to 320 °C at 40 °C/min, and held for 10 min.

To monitor the derivatization step and instrumental performance, a standard mix of *myo*- and *scyllo*-inositols with a known carbon isotopic composition (−37.2 and −36.9‰, respectively) was derivatized and the δ^13^C values were measured using GC–C–IRMS before and after each analytical run.

### 2.5 Data Expression

All of the δ^13^C and δ^15^N values are reported in relation to the known isotopic composition of the reference CO_2_ and N_2_ gases introduced into the ion source of IRMS at the beginning and end of each EA and GC run. All samples were measured 3 times, and the isotope ratio was expressed in δ‰ versus Vienna-Pee Dee Belemnite (V-PDB) for δ^13^C and atmospheric nitrogen for δ^15^N according to eq 1
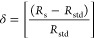
1where *R*_s_ is the isotope ratio of the sample and *R*_std_ is the isotope ratio of the internationally accepted standard.

The δ^13^C and δ^15^N values of pure non-derivatized proline and *myo*- and *scyllo*-inositols were determined by EA–IRMS. The isotopic values δ^13^C and δ^15^N were calculated against two working in-house standards (caseins), the first standard with δ^13^C = −21.98‰ and δ^15^N = 7.38‰ and the second standard with δ^13^C = −30.60‰ and δ^15^N = −3.40‰. They have themselves been calibrated against international reference materials: fuel oil NBS-22 with δ^13^C = −30.03‰, sucrose IAEA-CH-6 with δ^13^C = −10.45‰ [International Atomic Energy Agency (IAEA), Vienna, Austria], and l-glutamic acid USGS 40 with δ^13^C = −26.39‰ and δ^15^N = −4.52‰ (U.S. Geological Survey, Reston, VA, U.S.A.) for ^13^C/^12^C and ^15^N/^14^N and potassium nitrate IAEA-NO_3_ (δ^15^N = +4.7‰) from IAEA for ^15^N/^14^N.

The δ^15^N and δ^13^C values of proline in grape must were calculated against the standard proline, analyzed before and after each analytical run. The instrumental data were corrected on the basis of the difference between the δ^15^N and δ^13^C values of the standard proline in GC–C–IRMS (mean of six results, three before and three after the samples) and EA–IRMS, which was in any case always lower than 0.5 and 1.6‰ for δ^15^N and δ^13^C, respectively. Likewise, the δ^13^C value of *myo*- and *scyllo*-inositols in CRM was calculated against the mixture of standard *myo*- and *scyllo*-inositols, analyzed before and after each analytical run.

The effective δ^13^C values of proline and *myo*- and *scyllo*-inositols were obtained applying an empirical correction to remove the contribution of the derivatization reagents. The correction factor was calculated by determining the δ^13^C value of the underivatized standard (EA–IRMS) and the δ^13^C value of the derivatized standard (GC–C–IRMS)
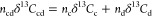
2where *n* is the number of moles of carbon and the subscripts c, d, and cd represent the compound of interest, the derivative group, and the derivatized compound, respectively.

### 2.6 Repeatability Limit and Uncertainty

One sample of grape must and one sample of CRM were treated and derivatized one time a month for 1 year to calculate the within-laboratory reproducibility standard deviation (SR) of δ^13^C and δ^15^N analysis of proline and *myo*- and *scyllo*-inositols. The analytical uncertainty (U) of δ^13^C and δ^15^N of proline and *myo*- and *scyllo*-inositol analysis, expressed as the coverage factor *k* = 2 multiplied for the SR was 0.4‰, whereas the reproducibility limit expressed as *k* × rad 2 × SR (with *k* = 2) was 0.6‰.^21^

To determine the repeatability limit for δ^13^C and δ^15^N of proline, 10 replicates of a grape must sample were derivatized, and each of them was analyzed using GC–C–IRMS. For δ^13^C of *myo*- and *scyllo*-inositols, a sample of CRM was considered. The standard deviation obtained (1σ) was 0.5‰ for δ^13^C of proline and 0.2‰ for all other parameters (Table S1 of the Supporting Information).

### 2.7 Statistical Analysis

The data were analyzed with the Statistica software 13.1 (StatSoft, Inc., Tulsa, OK, U.S.A.). Statistically significant correlations were verified using the Pearson correlation test. Statistically significant differences were observed using a Tukey honest significant difference (HSD) test. In all of the statistical analysis, the cutoff value was set at *p* < 0.05, which is associated with a significant difference between groups of values.

## 3 Results and Discussion

### 3.1 Study 1

In plants the sugar fraction (mainly glucose and fructose) is the result of photosynthesis that takes place in the green plastids of plant cells using carbon dioxide and water as precursors. Sugars, through glycolysis and the Krebs cycle, are used by the plants to synthesize 2-oxoglutarate that, thanks to the action of glutamate synthase, is converted in glutamate.^22^ Proline comes from glutamate, which is converted to proline by two consecutive reduction steps catalyzed first by pyrroline-5-carboxylate synthase (P5CS) and then by pyrroline-5-carboxylate reductase (P5CR).^23^ A strict correlation between the isotopic composition of sugars and amino acids is expected, given the biosynthetic path described above.

To investigate this relationship in real grape must samples we considered 59 authentic grape musts covering all of Italy and from two harvest years. The δ^13^C values of both sugar and proline ranged between −30 and −23‰, in line with the botanical origin of the matrix. Indeed the *Vitis vinifera* species belongs to plants with a C_3_ photosynthetic cycle and its δ^13^C normally ranges from −29 to −25‰.^30^

As reported in Table 1, it seems that there is not a big isotopic fractionation between the sugar fraction and proline. Δ^13^C (δ^13^C sugar – δ^13^C proline) varies in a narrow range between −1.7 and +1.6‰.

**Figure 1:**
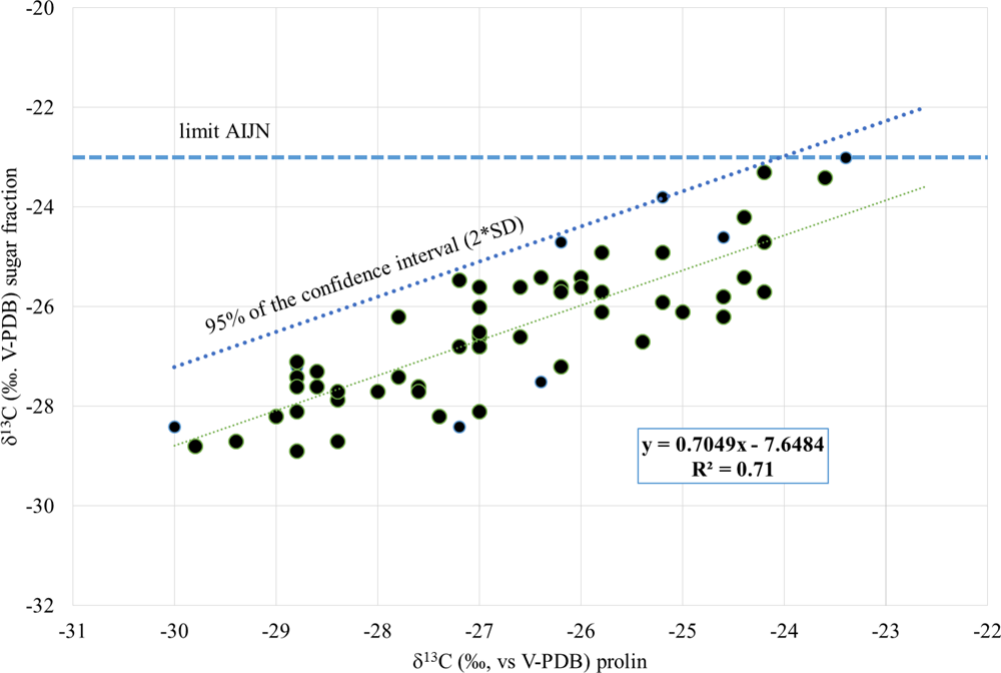
Correlation between δ^13^C of proline and δ^13^C of sugar fraction. The limit accepted by the Association of the Industries of Juices and Nectars from Fruits and Vegetables (AIJN) and the limit calculated on the basis of the 95% of the confidence intervals were reported as dotted lines.

By comparison of the δ^13^C values of the sugar fraction to the δ^13^C values of the amino acid proline we obtained a significant (*p* < 0.01; *R*^2^=0.71) linear relationship (δ^13^C_sugar_ = 0.70 × δ^13^C_proline_ – 7.65; Figure 1). We can define a threshold value for the relationship, calculating 95% of the confidence interval of the regression line from the following equation:
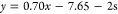
where “*y* = 0.70*x*–7.65” is the linear regression model obtained from the 59 data points, “2” is the Student *t*, and “s” is the standard deviation of the residues (difference between calculated and observed values), which in this case is 1.59.

Because the addition of exogenous cane sugar to grape must changes only δ^13^C of the sugar fraction and not that of proline, as here demonstrated (Table S2 of the Supporting Information), the fraudulent practice of sugar addition changes this relationship, which could go beyond the threshold value, even if δ^13^C of the sugar fraction is not outside the upper limit defined by the wine databank (EU Regulations 273 and 274/2018) and the natural grape variability [Guideline for Grape Juice of the Association of the Industries of Juices and Nectars from Fruits and Vegetables (AIJN)] and equal to −23‰.

**Figure 2:**
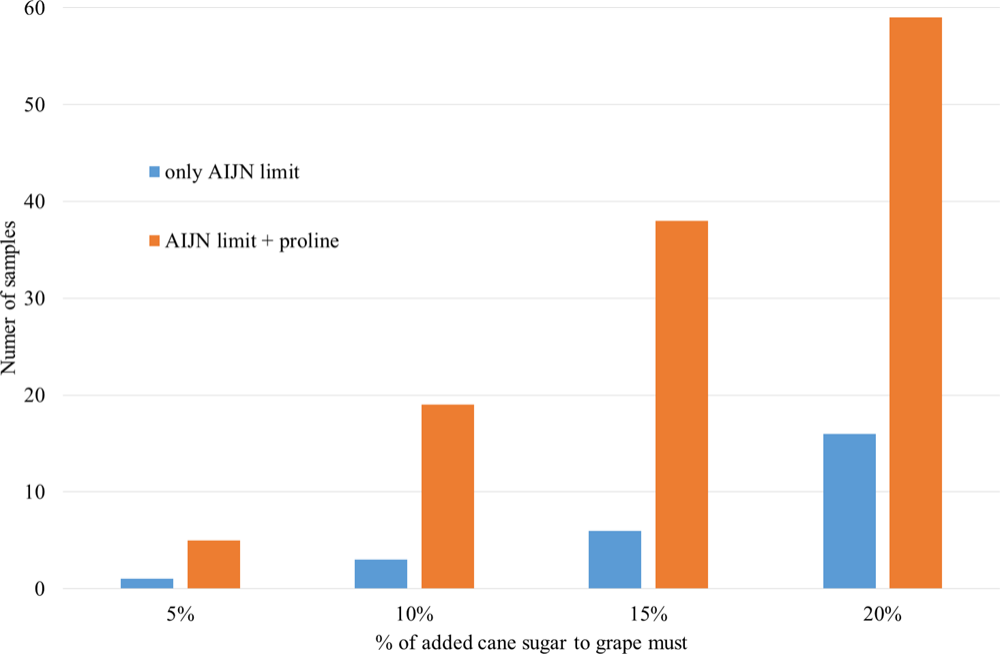
Improvement in grape must chaptalization detection by the number of samples identified as adulterated with cane sugar.

To demonstrate the possibility of improving chaptalization detection we simulated the adulteration of the 59 musts by adding an increasing percentage of cane sugar (δ^13^C = +12‰) and calculated the number of samples identified as adulterated with cane sugar, on the basis of both the δ^13^C value of the sugar fraction (which must be lower than −23‰) and that of the relationship between the two δ^13^C (Figure 2). In all cases the relationship improved detection of the chaptalization of grape must. With an addition of 20% cane sugar this new method made it possible to identify all of our adulterated samples as actually adulterated while, with an addition between 5 and 20%, the detection increases from a minimum of 6% (5 samples rather than 1 sample with 5% addition) to 72% (59 samples rather than 16 samples with 20% addition) (Figure 2).

δ^13^C analysis of both the sugar fraction and the amino acid proline and their correlation can therefore be considered as a reliable internal standard for improving detection of the fraudulent addition of sugar to must.

Our results can be used to check the authenticity of both grape must and wine. Indeed the proline utilization by *Saccharomyces cerevisiae* as a source of nitrogen requires the presence of oxygen and, therefore, in anaerobic fermentation conditions, as happens in wine production, proline is conserved in wine without isotopic fractionation.^8^ Moreover, δ^13^C of wine ethanol is strictly correlated with that of the relevant grape must sugar with a mean difference of 1.7‰ between them.^24^ Therefore, we can calculate both the regression line and 95% confidence limits also for δ^13^C of ethanol versus δ^13^C of proline in wine, by predicting δ^13^C of ethanol from δ^13^C of sugar (Figure 1).

**Figure 3:**
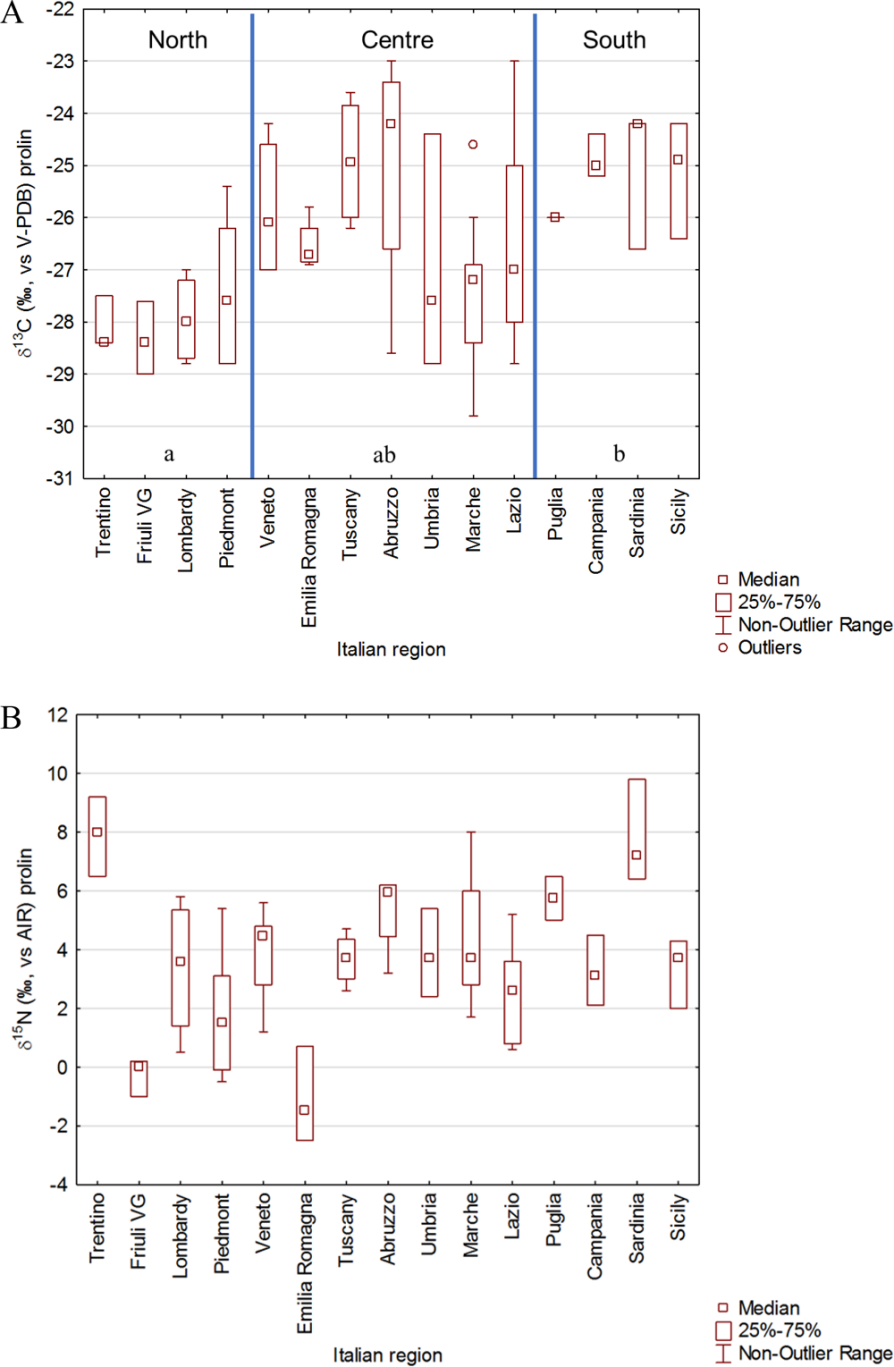
Geographical variability of (A) δ^13^C and (B) δ^15^N of proline in authentic Italian grape must.

The isotopic ratios of carbon (^13^C/^12^C, expressed as δ^13^C) and nitrogen (^15^N/^14^N, expressed as δ^15^N) were also analyzed in proline with the aim to evaluate the power of this marker to trace the geographical origin of grapes. We investigated δ^13^C of proline patterns in grape must across Italy (Figure 3A). Carbon isotope measurements described a gradient of more depleted values in the north of Italy to more enriched values in the south of Italy (Figure 3A). This was not surprising because stable carbon isotope ratios of plant materials are primarily related to the photosynthetic pathway used by a plant, even if δ^13^C in foodstuffs exhibits some geographical dependence linked to water stress and humidity during cultivation, although these differences are often very small in comparison to other isotopes.^25^

As reported in Figure 3B, it is not possible to identify a strict correlation between δ^15^N of proline and the region from which the sample originates. This could be due to the different agricultural practices adopted even within neighboring areas (e.g., organic or chemical fertilization) or the pedological characteristics of the soil. Indeed, inorganic fertilizers have δ^15^N values close to those of atmospheric N_2_ (from −6 to +6‰), whereas manure and other organic fertilizers can be substantially enriched (from +1 to +37‰).^26^

Paolini et al. reported that, despite nitrogen isotope fractionation through the chain soil–wine, the δ^15^N values of leaves, grapes, wine, and in particular proline in grape must and wine maintain the variability of δ^15^N in the growing soil.^8^ Samples from Sardinia have higher δ^15^N values (+9.8‰). This is in line with previous data reported for other matrices coming from this region, such as casein from pecorino cheese.^27^ The main reason could be the water stress as a result of the high temperature, which affects the biological turnover of nitrogen isotopes.^28^

### 3.2 Study 2

*myo*-Inositol, a carbocyclic sugar, is synthesized in grapes from glucose-6-phosphate (G-6-P) in two steps. First, an inositol-3-phosphate synthase enzyme (e.g., ISYNA1) isomerizes G-6-P to *myo*-inositol 1-phosphate, which is then finally dephosphorylated to give free *myo*-inositol by an inositol monophosphatase enzyme (e.g., IMPA1). It is normally abundant in must grape (more than 750 mg/kg of sugar) while its stereoisomer *scyllo*-inositol is less abundant (more than 38 mg/kg of sugar).^17,29^ In addition, a ratio of 20 or lower between *myo*- and *scyllo*-inositols has been suggested as an authenticity index.^17^ These limits of content are a useful routine control tool of CRM, but they can be easily falsified by fraudulent addition to the grape must of pure commercial polyalcohols in the right concentration. To describe the natural δ^13^C variability of these components several samples of authentic CRM were analyzed. In all of these samples the *myo*- and *scyllo*-inositol contents were measured and all of them fell within the limits suggested by Monetti et al. (Table 2).^17^

**Figure 4:**
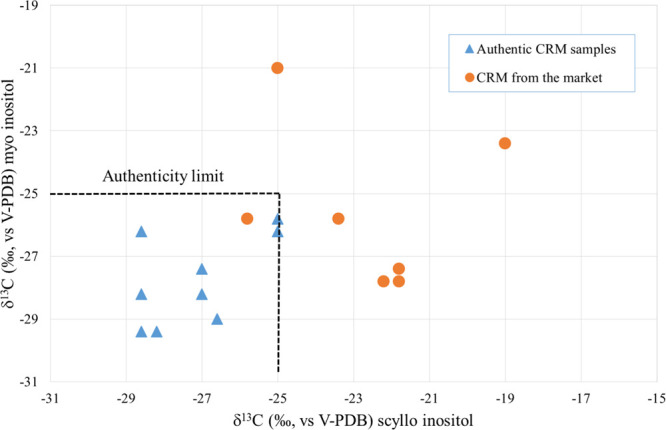
Variability of δ^13^C of *myo*- and *scyllo*-inositols of authentic CRM and samples collected on the market.

As reported in Table 2, *myo*- and *scyllo*-inositols that differ chemically only in the three-dimensional orientations of their atoms in space showed a similar δ^13^C range between −29.4 and −25.0‰, which is typical for plants with a C_3_ photosynthetic cycle.^30^ The maximum difference between the two δ^13^C is 2.4‰, and it can be used as a limit beyond which an addition of one of the two polyalcohols can be suspected. In addition, a limit of −25‰ for both the polyalcohols could be proposed (Figure 4). While commercial *myo*-inositol shows an average value of δ^13^C of −29.0 ± 0.3‰, probably as a result of the origin of the grapes from which the commercial product is extracted at a low cost, the δ^13^C value of *scyllo*-inositol is typical of C_4_ plants (−11.8 ± 0.3‰). *scyllo*-Inositol is rare and expensive, not being widely available from commercial sources, and for this reason it must be synthesized. Several synthetic approaches to produce *scyllo*-inositol are known.^31^ Rodriguez et al. present a concise synthesis of *scyllo*-inositol starting from inexpensive d-glucose.^32^

Commercial glucose is normally produced via the enzymatic hydrolysis of starch^33^ that belongs to the C_4_ plant, with a range of variability between −10 and −16‰,^30^ and this could justify the values found for *scyllo*-inositol. The illegal addition of this polyalcohol should be easily identified. A difference between the two δ^13^C, higher than 2.4‰ or a value of δ^13^C *scyllo*-inositol higher than −25‰, could be interpreted as an index of adulteration. Figure 4 shows the isotopic composition of *myo*- and *scyllo*-inositols of commercial CRM samples compared to the authentic sample. Only one sample falls within the variability limit, while five samples showed higher δ^13^C *scyllo*-inositol values (higher than −25‰). Sample Q (Table 2) is characterized by a high δ^13^C *myo*-inositol value. Maybe in this case commercial *myo*-inositol from a C_4_ plant source was used.

δ^13^C analysis of proline by GC–C–IRMS combined with the analysis of the sugar fraction represents a novel analytical tool to support and improve the detection of fraudulent addition of cane sugar to grape must and wine. δ^13^C of proline could be useful as geographical indicator, while δ^15^N of proline seems to correlate with the agronomic practices adopted. Moreover, the compound-specific analysis of δ^13^C of *myo*- and *scyllo*-inositols represents a useful tool to identify the illicit addition of these two polyalcohols in CRM obtained not from grape (e.g., from cereal) to mime the composition of an authentic CRM.

## Supporting Information Available

Map of sampling (Figure S1) (PDF)
Caption of Figure S1, repeatability of methods (Table S1), and effect of the addition of sugarcane to grape must on the δ^13^C and δ^15^N values of proline (Table S2) (PDF)

## Abbreviations Used

CRM: concentrated rectified grape must
GC: gas chromatography
IRMS: isotopic ratio mass spectrometry
