# Supplementary figures and images for: Gas Chromatography Combustion Isotope Ratio Mass Spectrometry for Improving the Detection of Authenticity of Grape Must
Source: J Agric Food Chem. 2020 Feb 3;68(11):3322–9. doi: 10.1021/acs.jafc.9b05952 (PMC7997364; doi:10.1021/acs.jafc.9b05952)

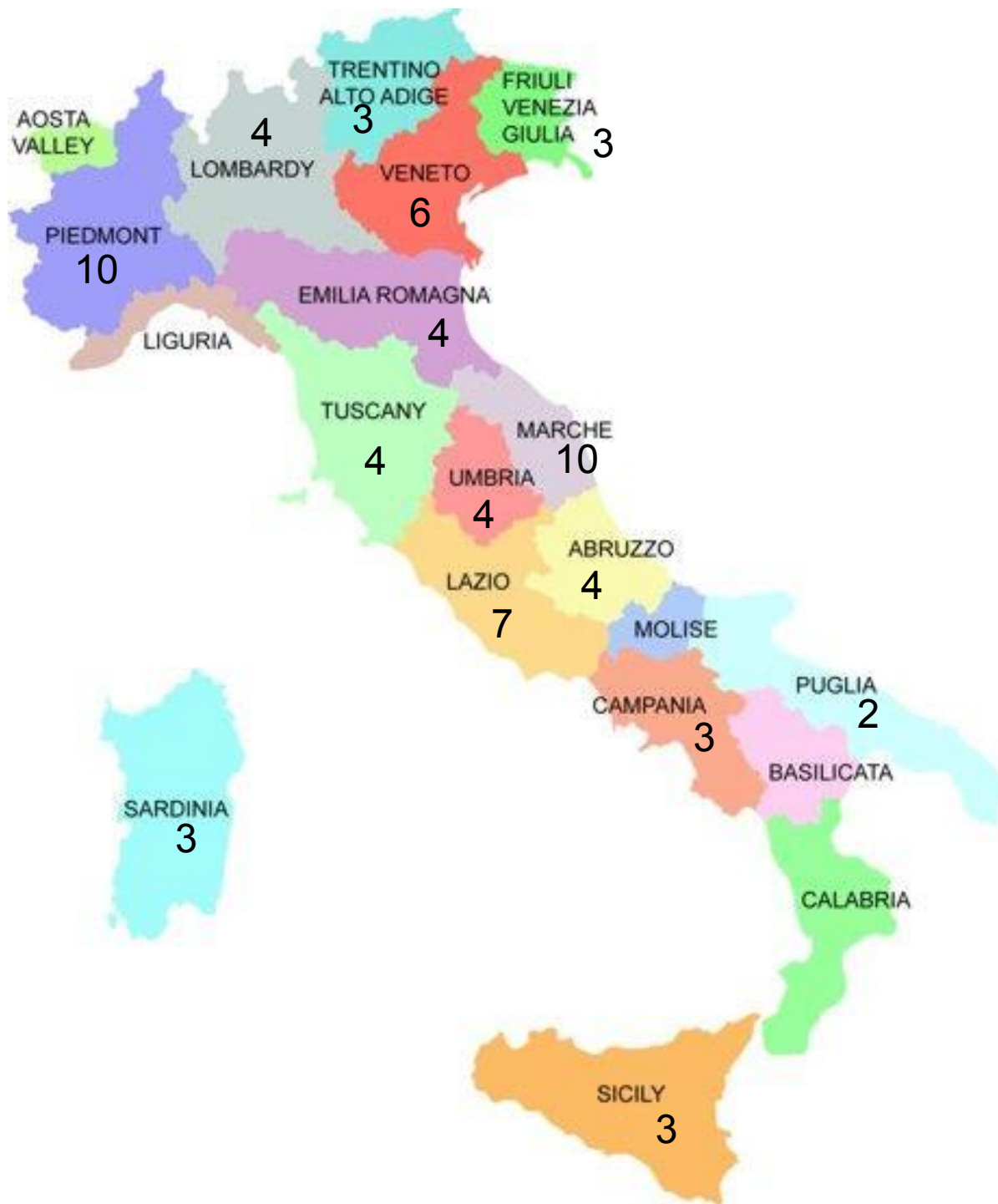

Supplement: Supplementary file 1 — jf9b05952_si_001.pdf [file jf9b05952_si_001.pdf]
