# Supplementary material for: Gas Chromatography Combustion Isotope Ratio Mass Spectrometry for Improving the Detection of Authenticity of Grape Must
Source: J Agric Food Chem. 2020 Feb 3;68(11):3322–9. doi: 10.1021/acs.jafc.9b05952 (PMC7997364; doi:10.1021/acs.jafc.9b05952)
Supplement: Supplementary file 2 — jf9b05952_si_002.pdf [file jf9b05952_si_002.pdf]

Supporting Information

Figure S1: Map of sampling (see a separate Supporting Information file for the graphic).

Table S1: Repeatability of Methods (δ^{13}C Data Corrected)

	δ^{13}C proline (‰, vs V-PDB)	δ^{15}N proline (‰, vs AIR)	δ^{13}C scyllo inositol (‰. vs V-PDB)	δ^{13}C myo-inositol (‰. vs V-PDB)
1	-24.4	5.3	-28.6	-29.0
2	-25.4	5.5	-28.7	-29.1
3	-25.4	5.2	-29.0	-29.0
4	-25.0	5.3	-28.5	-28.6
5	-24.4	5.3	-28.5	-28.6
6	-24.4	5.7	-29.0	-28.5
7	-25.4	5.2	-28.6	-28.7
8	-24.8	5.3	-28.2	-28.7
9	-25.0	5.4	-28.5	-29.0
10	-25.8	5.5	-28.6	-29.0
**Mean**	**-25.0**	**5.4**	**-28.6**	**-28.8**
**SD**	**0.5**	**0.2**	**0.2**	**0.2**

Table S2: Effect of the Addition of Sugarcane to Grape Must on the δ^{13}C and δ^{15}N Values of Proline

% w/w of cane added to must	δ^{13}C proline (‰, vs V-PDB)	δ^{15}N proline (‰, vs AIR)
0	-24.4	5.4
14	-25.0	5.3
28	-24.4	5.5
32	-24.4	5.2
